# Protective Ventilation of Preterm Lambs Exposed to Acute Chorioamnionitis Does Not Reduce Ventilation-Induced Lung or Brain Injury

**DOI:** 10.1371/journal.pone.0112402

**Published:** 2014-11-07

**Authors:** Samantha K. Barton, Timothy J. M. Moss, Stuart B. Hooper, Kelly J. Crossley, Andrew W. Gill, Martin Kluckow, Valerie Zahra, Flora Y. Wong, Gerhard Pichler, Robert Galinsky, Suzanne L. Miller, Mary Tolcos, Graeme R. Polglase

**Affiliations:** 1 The Ritchie Centre, MIMR-PHI Institute of Medical Research, Monash University, Clayton, Victoria, 3168, Australia; 2 Department of Obstetrics and Gynecology, Monash University, Clayton, Victoria, 3168, Australia; 3 School of Women's and Infants' Health, The University of Western Australia, Crawley, Western Australia, 6009, Australia; 4 Department of Neonatal Medicine, Royal North Shore Hospital and University of Sydney, Sydney, New South Wales, 2065, Australia; 5 Department of Pediatrics, Medical University Graz, Auenbruggerplatz 30, Graz, Austria, 8036; Hôpital Robert Debré, France

## Abstract

**Background:**

The onset of mechanical ventilation is a critical time for the initiation of cerebral white matter (WM) injury in preterm neonates, particularly if they are inadvertently exposed to high tidal volumes (V_T_) in the delivery room. Protective ventilation strategies at birth reduce ventilation-induced lung and brain inflammation and injury, however its efficacy in a compromised newborn is not known. Chorioamnionitis is a common antecedent of preterm birth, and increases the risk and severity of WM injury. We investigated the effects of high V_T_ ventilation, after chorioamnionitis, on preterm lung and WM inflammation and injury, and whether a protective ventilation strategy could mitigate the response.

**Methods:**

Pregnant ewes (n = 18) received intra-amniotic lipopolysaccharide (LPS) 2 days before delivery, instrumentation and ventilation at 127±1 days gestation. Lambs were either immediately euthanased and used as unventilated controls (LPS_UVC_; n = 6), or were ventilated using an injurious high V_T_ strategy (LPS_INJ_; n = 5) or a protective ventilation strategy (LPS_PROT_; n = 7) for a total of 90 min. Mean arterial pressure, heart rate and cerebral haemodynamics and oxygenation were measured continuously. Lungs and brains underwent molecular and histological assessment of inflammation and injury.

**Results:**

LPS_INJ_ lambs had poorer oxygenation than LPS_PROT_ lambs. Ventilation requirements and cardiopulmonary and systemic haemodynamics were not different between ventilation strategies. Compared to unventilated lambs, LPS_INJ_ and LPS_PROT_ lambs had increases in pro-inflammatory cytokine expression within the lungs and brain, and increased astrogliosis (p<0.02) and cell death (p<0.05) in the WM, which were equivalent in magnitude between groups.

**Conclusions:**

Ventilation after acute chorioamnionitis, irrespective of strategy used, increases haemodynamic instability and lung and cerebral inflammation and injury. Mechanical ventilation is a potential contributor to WM injury in infants exposed to chorioamnionitis.

## Introduction

Cerebral white matter (WM) injury is common in preterm infants, and can result from a multitude of insults during pregnancy and after birth [1]. Ventilation onset after preterm birth in lambs can instigate an injurious cascade resulting in ventilation-induced brain injury, particularly if high tidal volumes (V_T_) are used [2]. This is especially relevant given that up to 80% of preterm infants inadvertently receive high V_T_ ventilation in the delivery room due to the limitations of the devices used [3]–[6]. The major mechanisms leading to ventilation-induced brain injury include: 1) altered pulmonary blood flow, leading to adverse cardiac output and consequent abnormal changes to cerebral blood flow [2] and 2) the initiation of a profound pulmonary inflammatory response [7]–[9] that initiates a systemic inflammatory cascade [10]–[12] resulting in a localized inflammatory response within the cerebral WM [2], [13]. These mechanisms are consistent with previously identified pathways of brain injury in preterm infants [14]. Importantly, these pathways highlight the critical interaction between the lungs, heart and brain in the progression of preterm brain injury during the immediate transition at birth. Importantly, improving the initial ventilation strategy at birth mitigates ventilation-induced brain injury in otherwise healthy preterm lambs [2]. This relationship has not been investigated in prenatally compromised models, such as preterm lambs exposed to intrauterine inflammation.

Intrauterine inflammation, diagnosed clinically as chorioamnionitis, affects almost 10% of pregnancies [15] with the incidence inversely proportional to gestational age [16]; up to two-thirds of extremely preterm infants are exposed to chorioamnionitis [16]. Chorioamnionitis impairs development and causes gross injury to organs such as the lungs and brain [17]–[20], and is associated with an increased risk and severity of intraventricular haemorrhage and diffuse WM injury in preterm infants. Furthermore, preterm infants exposed to chorioamnionitis are at an increased risk of developing cerebral palsy (4-fold) [20], [21] and schizophrenia (16-fold) [22] later in life. Studies have demonstrated that intrauterine inflammation induces a profound pulmonary [23], [24], systemic [12], [25] and cerebral inflammatory response [26]. Further, as intrauterine inflammation in of itself can alter fetal cerebral haemodynamics [27] and increase the prevalence of impaired cerebral autoregulation [28], the mechanisms of inflammation-induced brain injury are consistent with that of perinatal brain injury. However, few studies have investigated the consequences of mechanical ventilation on lung and brain inflammation and injury after chorioamnionitis.

We investigated whether the initiation of ventilation exacerbates inflammation and injury of the lungs and brain after intrauterine inflammation induced by intra-amniotic lipopolysaccharide (LPS) injection [29] two days prior to delivery. We hypothesized that high V_T_ ventilation after acute intrauterine inflammation would exacerbate lung and cerebral white matter inflammation and injury, and a protective ventilation strategy would reduce this injury.

## Materials and Methods

### Ethics Statement

The experimental protocol was approved by the Monash Medical Centre ‘A’ (MMCA) animal ethics committee and was conducted according to guidelines established by the National Health and Medical Research Council of Australia.

### Instrumentation and Delivery

Ultrasound guided intra-amniotic (IA) injection of LPS (10 mg; from *Escherichia coli* 055: B5; Sigma-Aldrich, Australia) was administered at 125±1 days (d) of gestation (term ∼147 d; n = 18). At 127±1 d, unventilated controls, which received IA LPS (LPS_UVC_; n = 6), were humanely killed (sodium pentobarbitone: >100 mg/kg i.v.) without surgical instrumentation, anaesthesia or ventilation. For the ventilated groups (all exposed to LPS), the ewe and fetus were anesthetized with isoflurane (1.5–3.0% in 100% oxygen, Bomac Animal Health, NSW, Australia). The fetal head and neck were exposed via caesarean section and ultrasonic flow probes (Transonic Size 3PS, ADInstruments, Bella Vista, Australia) were placed around the left and right carotid arteries for constant monitoring of carotid blood flows, which correlate closely with cerebral blood flow [30]. Lambs were delivered, dried, weighed and placed under a radiant heater. Polyvinyl catheters (ID 0.86 mm, OD 1.52 mm, Dural Plastics, Australia) were placed in an umbilical artery and vein for mean arterial pressure monitoring and anaesthesia maintenance, respectively. The lambs were sedated (Alfaxane i.v. 5–15 mg/kg/h; Jurox, East Tamaki, Auckland, New Zealand) throughout the experiment to minimize spontaneous breathing. Ewes were humanely killed (sodium pentobarbitone: >100 mg/kg i.v.) after delivery.

### Ventilation strategy

Prior to experiment commencement, we randomly allocated each ewe's identification number to a ventilation protocol to avoid any bias. LPS-exposed lambs received either an injurious or protective ventilation strategy as described previously [2]. Briefly, injuriously ventilated lambs (LPS_INJ_; n = 5) received a high V_T_ targeting 12 ml/kg for the initial 15 min using volume guarantee ventilation (Babylog 8000+, Dräger, Lübeck, Germany) with peak inflation pressure (PIP) limited to 50 cmH_2_O. After the initial 15 min, lambs V_T_ was reduced to 7 ml/kg for the remaining 75 min. The protective ventilation group (LPS_PROT_; n = 7) received prophylactic surfactant (100 mg/kg, Curosurf, Chiesi Pharma, Italy), a 20-second sustained inflation (Neopuff; Fisher & Paykel Healthcare, Panmure, Auckland New Zealand) with a PIP of 30 cmH_2_O, followed by ventilation with V_T_ 7 mL/kg for 90 min. Throughout ventilation, positive end-expiratory pressure (PEEP) was 5 cmH_2_O, inspiratory time was 0.3 s and expiratory time was 0.6 s. Lambs were ventilated with warm humidified air. The fraction of inspired oxygen was set initially at 0.4 but was subsequently altered to maintain arterial oxygen saturation (Masimo, Irvine, CA) within 88–95%. Lamb well-being was monitored throughout via frequent blood gas samples (ABL30, Radiometer, Copenhagen, Denmark).

### Haemodynamic Measurements

Carotid blood flows and mean arterial pressure (DTX Plus Transducer; Becton Dickinson, Singapore) were recorded continuously (Powerlab; ADInstruments, Castle Hill, NSW, Australia). Doppler ultrasound was performed regularly to monitor left ventricular output and the ratio of right-to-left/left-to-right blood flow through the ductus arteriosus as described previously [2]. Spatially resolved spectroscopy (SRS, NIRO 200 Spectrophotometer; Hamamatsu Photonics K.K., Hamatsu City, Japan) was used to continuously measure mixed cerebral oxygen saturation expressed as tissue oxygenation index (TOI, %), with optodes placed 4 cm apart on the scalp at the parietal region. The change in concentrations (µM.cm) of total haemoglobin were measured and used to determine change in cerebral blood volume (ΔCBV; mL/100 g of brain tissue) using differential pathlength factor of 4.99 [31], [32] and the equation ΔCBV =  (ΔHbT · MW_haemoglobin_ · 10^−6^)/(tHb · 10^−2^×CLVHR · Dt · 10) where ΔHbT =  change in total haemoglobin in µM.cm, MW_haemoglobin_ =  molecular weight of haemoglobin (64,500), tHb =  concentration of haemoglobin in large vessels in g · 100 mL^−1^, CLVHR =  cerebral to large vessel haematocrit ratio (0.69), and Dt =  brain tissue density in g · mL^−1^ (1.05).

### Physiological Calculations

Ventilator efficiency index was calculated as VEI = 3800/(PIP – PEEP) · rate · PaCO_2_ [33]. Mean airway pressure was calculated as P_AW_ =  (PIP – PEEP) · 0.6/1.5+PEEP. Oxygenation index was calculated as OI =  (F_i_O_2_ · P_AW_)/PaCO_2_. Dynamic lung compliance was calculated as lung compliance  =  (V_T_/body weight)/(PIP – PEEP). Cerebral oxygen delivery (mL/kg/min) was calculated as DO_2_ =  total carotid blood flow (CBF) · ((Hb (g/dL)/10 · SaO_2_/100)/100) · 1.36+ (0.003 · PaO_2_). Cerebral oxygen extraction was calculated as cerebral oxygen extraction  =  (SaO_2_ – TOI)/SaO_2_) [2], [34], [35].

### Tissue Collection

At the end of the study, preterm lambs (127±1 d) were humanely killed with an overdose of sodium pentobarbitone (100 mg/kg i.v.) and their lungs and brains were excised. The lungs were dissected and the right lower lobe was snap frozen and RNA was extracted for quantitative real-time PCR (qRT-PCR). The brain was halved along the midline and each hemisphere was further dissected coronally into 5 mm-thick blocks. Blocks from the left cerebral hemisphere were snap frozen in liquid nitrogen individually, while those from the right cerebral hemisphere were immersion fixed in 4% paraformaldehyde in 0.1 M phosphate buffer (pH 7.4), processed through alcohol and xylene washes and embedded in paraffin wax. Equivalent blocks, at the level of the lateral ventricle in the parietal lobe were chosen from the left and right hemisphere of each animal for subsequent molecular and immunohistochemical analysis. These blocks were chosen as they contain both periventricular and subcortical WM.

### Molecular Assessment of the Lung and Brain

Lung and brain tissue frozen in liquid nitrogen was homogenized and total RNA was isolated (RNeasy Maxi Kit, Qiagen) and reverse-transcribed into cDNA (SuperScript III reverse transcriptase, Invitrogen). Brain tissue was a mixture of white and grey matter including regions from the periventricular WM, subcortical WM, cortical grey matter, external capsule and striatum. Relative mRNA expression of key pro-inflammatory interleukins (*IL-1β, IL-6* and *IL-8;* lung and brain) were measured by qRT-PCR (see table 1 for primer sequences) using Applied Biosystems 7900HT Fast Real-Time PCR system. The expression of all genes was normalized to the *18S* rRNA for each sample using the cycle threshold (ΔC_T_) method of analysis and was expressed relative to the LPS_UVC_ group.

**Table 1:** Primer Sequences for real-time PCR.

Gene	Species	Accession No.	Primer Sequence	Amplicon Length, nt
18S	Rat	X01117	5′-GTAACCCGTTGAACCCCATT-3′	105
			5′-CCATCCAATCGGTAGTAGCG-3′	
IL-1β	Sheep	NM_001009465	5′-CGATGAGCTTCTGTGTGATG-3′	120
			5′-CTGTGAGAGGAGGTGGAGAG-3′	
IL-6	Sheep	NM_001009392	5′-CGCAAAGGTTATCATCATCC-3′	107
			5′-CCCAGGAACTACCACAATCA-3′	
IL-8	Sheep	NM_001009401	5′-CCTCAGTAAAGATGCCAATGA-3′	82
			5′-TGACAACCCTACACCAGACC-3′	

### Brain Histology and Immunohistochemistry

Serial sections (10 µm thick) were stained in duplicate with the following antibodies: rabbit anti-ionized calcium-binding adapter molecule-1 (Iba-1; 1∶1500, WAKO Pure Chemical Industries, Osaka, Japan) to identify ramified and amoeboid microglia, rabbit serum albumin (1∶1000, Accurate Chemical & Scientific Corporation, USA) to assess blood brain barrier permeability, and rabbit anti-glial fibrillary acidic protein (GFAP; 1∶1000, DAKO, Glostrup, Denmark) to identify reactive astrocytes. Before incubation with anti-Iba-1 and –GFAP, sections were pretreated with citrate buffer (pH 6.0) in a microwave oven. All sections were incubated with appropriate biotinylated secondary antibodies (Iba-1, 1∶200 for 90 mins; serum albumin and GFAP, 1∶200 for 60 mins). Sections were reacted using the Vectastain Elite ABC kit (Iba-1, GFAP: 1∶1∶200; 90 mins; Vector Laboratories, Burlingame, CA) or with streptavidin horseradish peroxidase (serum albumin: 1∶200, 30 mins; GE Healthcare). The Iba-1-immunostained sections were counterstained with 0.1% thionin in acetate buffer (pH 4.5). Duplicate sections were stained with DeadEnd Colorimetric TUNEL System (Promega, Madison, WI, USA) to detect DNA fragmentation as an indication of cell death. For each antibody, sections from each cohort were simultaneously reacted to reduced staining variability. There was no staining when the primary antibody was omitted.

### Quantitative Analysis

Analysis was conducted on coded slides (observer blinded to the treatment) using ImageJ (Iba-1, GFAP; NIH image, Bethasda, Maryland, USA) or ImageScope (TUNEL: Aperio Technologies, California, USA). We quantified GFAP-immunoreactive astrocytes and Iba-1 immunoreactive microglia in 2–6 random non-overlapping fields from both the periventricular and subcortical WM, with a total of 8 fields per section. We morphologically distinguished ramified microglia (presence of long branching processes) and amoeboid microglia (large, densely stained soma with retracted processes) [36] and expressed the findings as total microglia and percentage of amoeboid to total microglia. Cell densities are expressed as cells/mm^2^. All TUNEL-positive cells were counted in periventricular and subcortical WM and expressed as numbers of cells per area of WM measured (cell/mm^2^). The total number of vessel profiles with serum albumin extravasation within the WM were counted and expressed as number of leaky vessels per WM area. Mean values for each parameter were then calculated for each treatment group.

### Statistical Analysis

Serial physiological data were compared between ventilated groups using two-way repeated measures ANOVA (Sigmaplot, Systat Software Inc). The initial 15 min ventilation period and the entire 90 min ventilation period were compared in two separate analyses. Post-hoc comparisons were conducted using the Holm-Sidak method. The effect of ventilation strategy was determined by comparing LPS_UVC_, LPS_PROT_ and LPS_INJ_ groups using one-way ANOVA (for parametric data) with Dunnett's test used for post-hoc comparisons (with LPS_UVC_ as control). For non-parametric data, Kruskal-wallis ANOVA on Ranks was used with the Dunn test for post-hoc comparisons. Data are presented as mean ±SEM. Values of p<0.05 were considered statistically significant.

## Results

Successful injection of LPS into the amniotic sac was confirmed by electrolyte analysis of amniotic fluid aspirated at the time of injection [37]. The presence of intrauterine inflammation was confirmed visually in all LPS lambs by the presence of thickened and edematous fetal membranes characteristic of this experimental intervention [37].

Fetal blood gases, body weights and sex of the lambs were not different between LPS exposed unventilated controls and ventilated groups (average between groups: pH = 7.27±0.03, PaCO2 (mmHg)  = 53.23±3.96, PaO2 (mmHg)  = 41.13±5.79, SaO2 (%)  = 81.38±7.13, body weight (kg)  = 3.36±0.15, male (n)  = 10).

### Ventilation and Oxygenation

**Figure 1:**
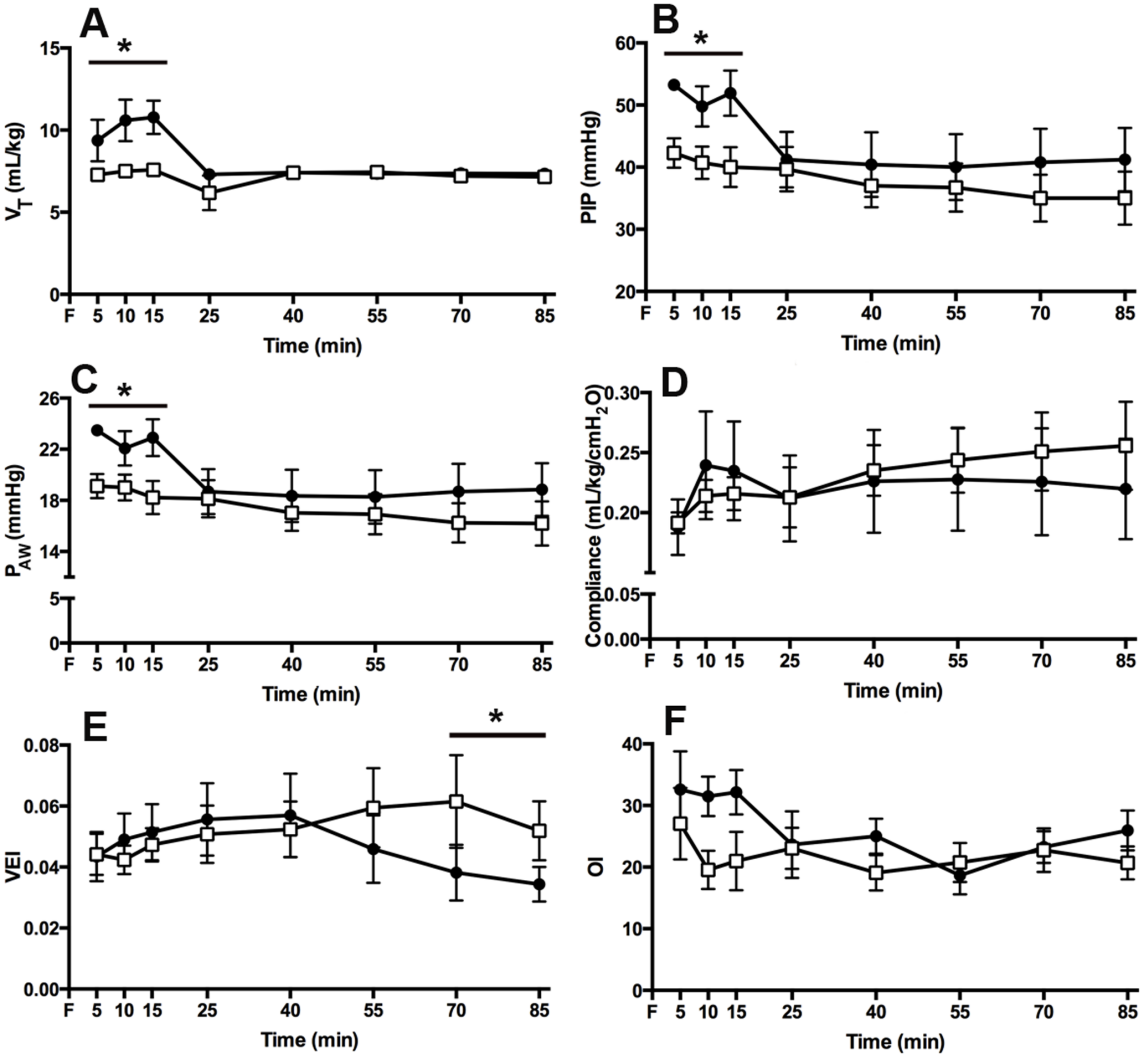
Ventilation Parameters and Oxygenation. (A) Tidal volume delivered to LPS_PROT_ lambs (open squares) and LPS_INJ_ lambs (closed circled). (B) Peak inspiratory pressure delivered. (C) Mean airway pressure in both groups. (D) Dynamic lung compliance in both groups. (D) Ventilation efficiency index and (E) Oxygenation Index in both groups with a higher index correlating to poorer oxygenation. * p<0.05.

The LPS_INJ_ group received a higher average V_T_ during the initial 15 min ventilation period than the LPS_PROT_ group (average V_T_: 10.3±0.6 vs. 7.5±0.2 mL/kg; p = 0.009; Fig. 1A) resulting in a higher minute volume (p = 0.034); no differences in V_T_ or minute volume were present after the initial 15 min ventilation period. PIP (Fig. 1B) and P_aw_ (Fig. 1C) were higher in the LPS_INJ_ group than the LPS_PROT_ group during the initial 15 min (p = 0.040 and p = 0.047, respectively) but not thereafter. Dynamic lung compliance (Fig. 1D) was not different between groups, but VEI (p = 0.036; Fig. 1E) was higher (better) in LPS_PROT_ lambs from 70 minutes. Oxygenation index was higher (indicating worse oxygenation) in LPS_INJ_ lambs compared to LPS_PROT_ lambs at 10 and 15 min (p = 0.09; Fig. 1F) but was not different thereafter. PaCO_2_, PaO_2_ and FiO_2_ were not different between groups at any time (data not shown).

### Cardiopulmonary and Cerebral Haemodynamics

**Figure 2:**
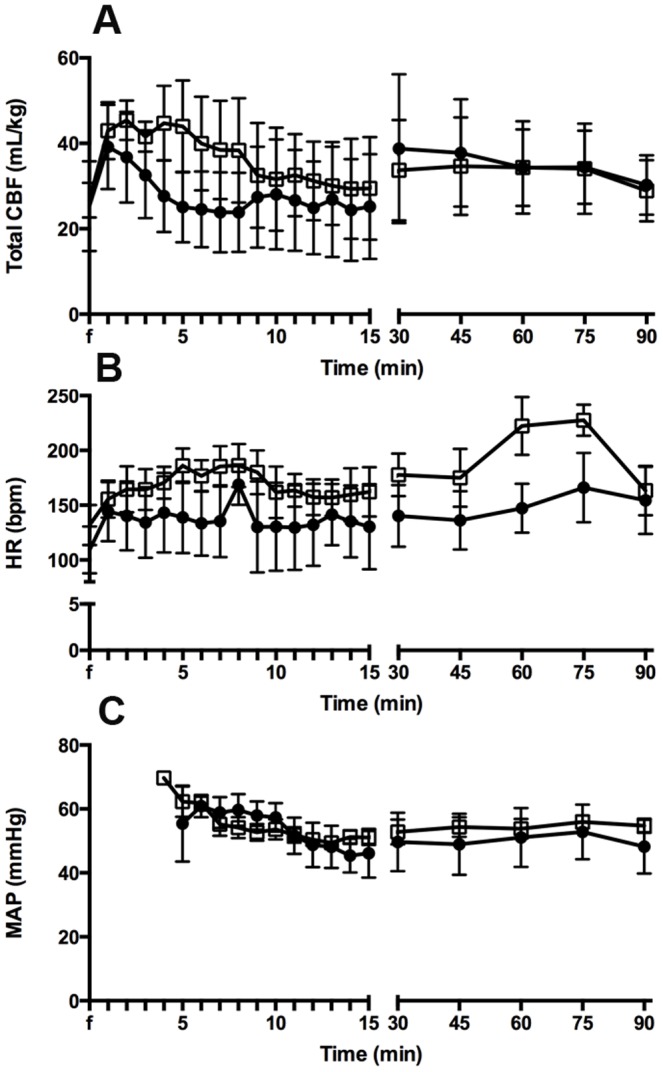
Systemic and Cerebral Haemodynamics. (A) Total carotid blood flow, (B) heart rate and (C) mean arterial pressure in the LPS_PROT_ group (open squares) and LPS_INJ_ group (closed circles).

Total CBF (Fig. 2A) was highly variable in both LPS_INJ_ and LPS_PROT_ groups, with evidence of rapid fluctuations in CBF in both groups. Doppler echocardiography measurements of right-to-left to left-to-right blood flow through the ductus arteriosus and left ventricular output were not different between groups, but a decrease in left ventricular output was observed over time in both groups (p<0.001; data not shown). Heart rate (Fig. 2B) and mean arterial pressure (Fig. 2C) were not different between ventilation groups.

### Cerebral oxygenation

**Figure 3:**
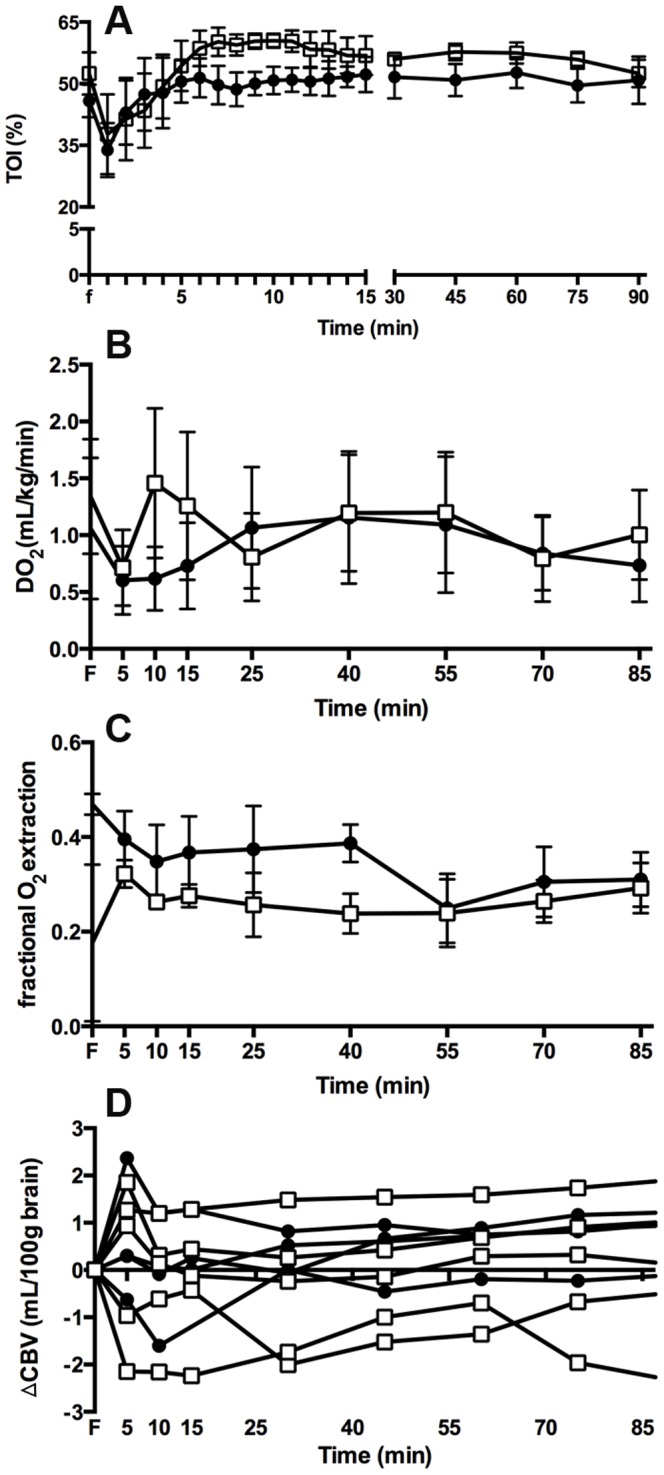
Cerebral Oxygenation. (A) Tissue oxygenation index, (B) cerebral oxygen delivery, (C) cerebral oxygen extraction and (D) cerebral blood volume in the LPS_PROT_ group (open squares) and LPS_INJ_ group (closed circles).

Tissue oxygenation index was not different between groups (Fig. 3A). Cerebral oxygen delivery (Fig. 3B) was not different between ventilation groups, however cerebral oxygen extraction tended to be higher in the LPS_INJ_ lambs compared to LPS_PROT_ lambs (p = 0.057; Fig. 3C). Changes in cerebral blood volume were not different between groups (Fig. 3D).

### Pulmonary Inflammation

**Figure 4:**
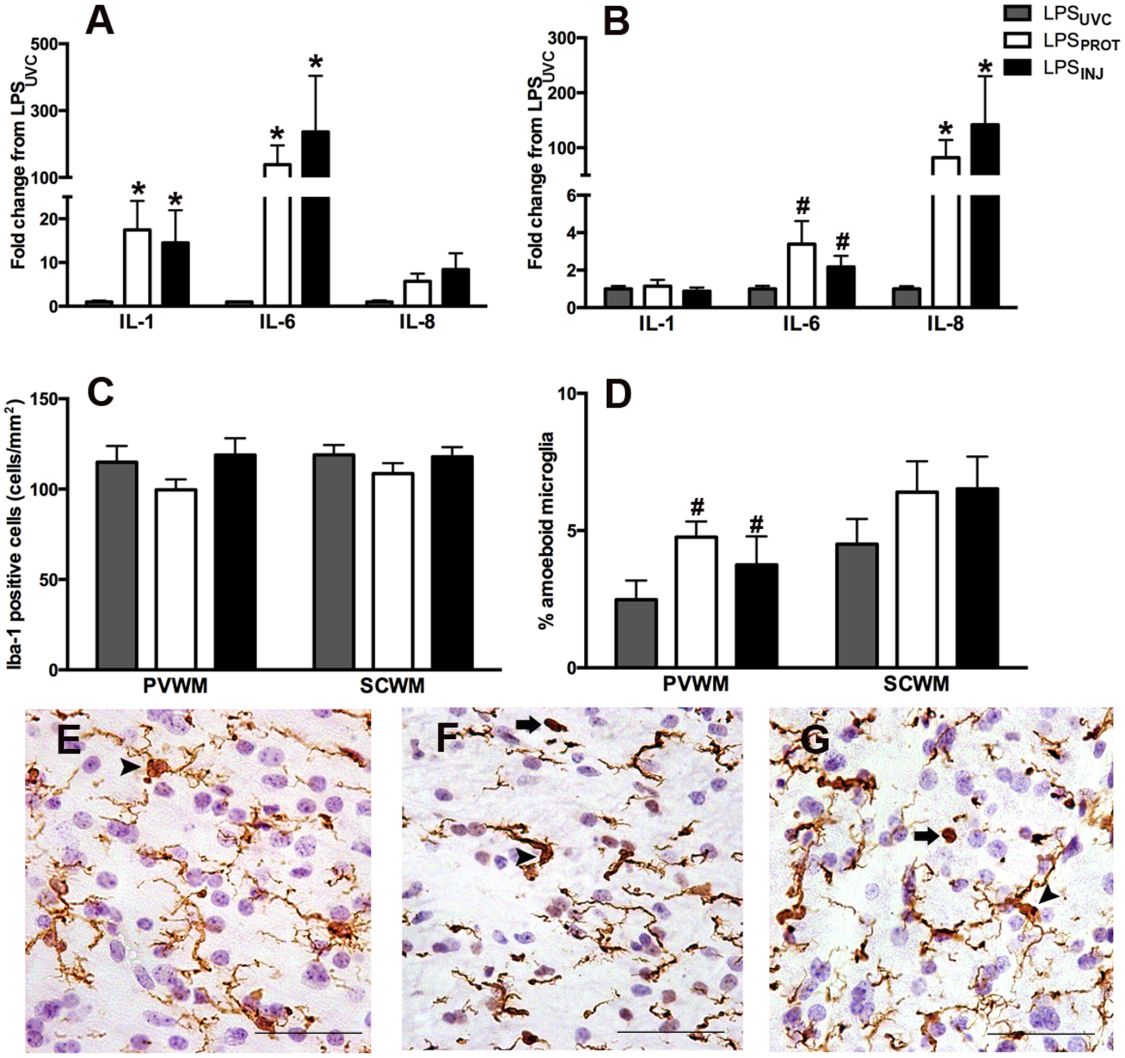
Lung and Cerebral White Matter Inflammation. Pro-inflammatory cytokine gene expression of interleukin (IL)-1β, IL-6 and IL-8 in the lung (A) and brain (B) as a fold change in the LPS_PROT_ and LPS_INJ_ groups from the LPS_UVC_ group. (C) The total number of Iba-1 positive cells in the periventricular (E–G; LPS_UVC_, LPS_PROT_ and LPS_INJ_, respectively) and subcortical white matter. (D) The percentage of amoeboid to total microglia in the periventricular (E–G; arrowheads indicate ramified microglia and arrows indicate amoeboid microglia) and subcortical white matter. Grey bars denote the LPS_UVC_ group, white bars denote LPS_PROT_ and black bars denote LPS_INJ_. Scale bar represents 50 µm. * p<0.05. # p<0.1.

Lung IL-1β and IL-6 mRNA levels were higher in ventilated groups compared to LPS_UVC_ lambs (p = 0.041 and p<0.001 respectively), but there was no difference between LPS_INJ_ and LPS_PROT_ groups (Fig. 4A). Lung IL-8 mRNA expression was not different between groups (Fig. 4A).

### Molecular Assessment of Cerebral Inflammation and injury

Cerebral IL-1β was not different between LPS_UVC_, LPS_INJ_ and LPS_PROT_ groups (Fig. 4B); IL-6 mRNA expression was higher after ventilation but did not reach significance (p = 0.088); no difference between ventilation strategies was observed (Fig. 4B). Cerebral IL-8 mRNA expression was significantly higher in the LPS_PROT_ and LPS_INJ_ lambs (p<0.001 for both) compared to LPS_UVC_ lambs but there was no difference between ventilation groups (Fig. 4B).

### Histological Assessment of Cerebral Inflammation and Injury

The density of Iba-1-positive cells (Fig. 4C) in the periventricular WM (Fig. 4E–G) or subcortical WM were not different between LPS_UVC_, LPS_INJ_ and LPS_PROT_ groups. The percentage of amoeboid microglia was not different between groups in the subcortical WM and tended higher in the periventricular WM (Fig. 4D–G) in the LPS_PROT_ and LPS_INJ_ groups compared to LPS_UVC_ (p = 0.081).

**Figure 5:**
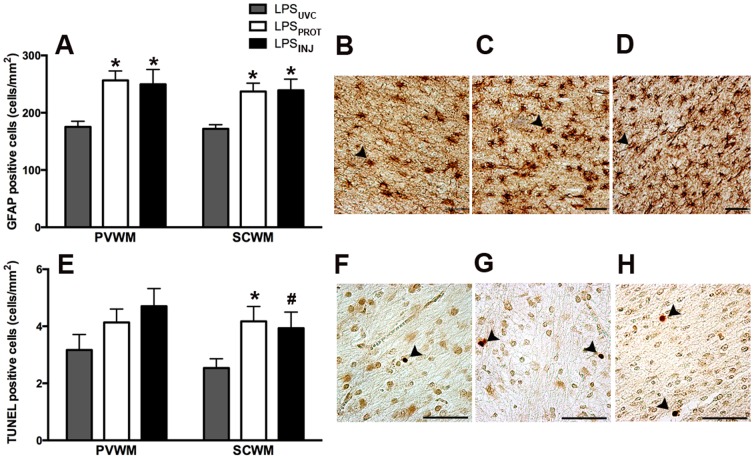
Cerebral White Matter Injury and Gliosis. (A) GFAP-positive astrocytes in the periventricular and subcortical (B–D; LPS_UVC_, LPS_PROT_, LPS_INJ_, respectively) white matter. (E) TUNEL-positive cells in the periventricular and subcortical (F–H; LPS_UVC_, LPS_PROT_, LPS_INJ_, respectively) white matter. Grey bars denote the LPS_UVC_ group, white bars denote LPS_PROT_ and black bars denote LPS_INJ_. Scale bar represents 50 µm. * p<0.05. # p<0.1.

**Figure 6:**
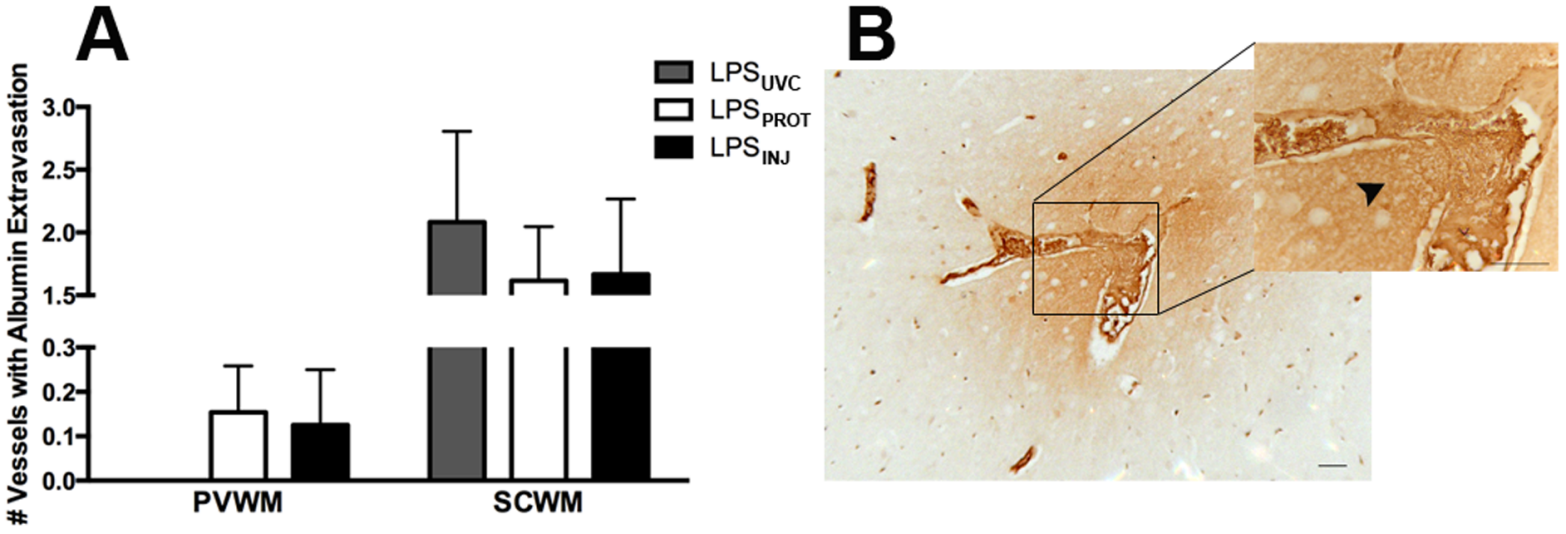
Vascular Leakage. (A) The number of vessel profiles with albumin extravasation in the periventricular and subcortical white matter. (B) A representative image of albumin extravasation indicated by the black arrowhead; a vessel within the subcortical white matter of an LPS_PROT_ lamb. Grey bars denote the LPS_UVC_ group, white bars denote LPS_PROT_ and black bars denote LPS_INJ_. Scale bar represents 20 µm.

The density of GFAP-positive astrocytes in the periventricular (Fig. 5A) and subcortical WM (Fig. 5A-D) was significantly higher in LPS_PROT_ and LPS_INJ_ groups compared to LPS_UVC_ lambs but there was no difference between LPS_PROT_ and LPS_INJ_ groups. The density of TUNEL-positive cells in the subcortical WM was significantly higher in the LPS_PROT_ lambs compared to LPS_UVC_ lambs (p = 0.033), but was not higher in LPS_INJ_ lambs (p = 0.097; Fig. 5E–H); there was no difference between LPS_PROT_ and LPS_INJ_ groups. In the periventricular WM, there was no difference in the density of TUNEL-positive cells between groups (Fig. 5E). The number of vessels with albumin extravasation was not different between groups (Fig. 6) in the periventricular WM (p = 0.47) or subcortical WM (p = 0.82).

## Discussion

The initiation of ventilation of preterm newborns increases lung and brain inflammation and injury, particularly if high tidal volumes are used [2], [38]. We have now shown that ventilation after IA LPS exposure increased pro-inflammatory cytokine mRNA levels in the lungs and brain and increased the density of ameoboid microglia, astrocytes and apoptotic cells in a WM region specific manner (i.e. periventricular vs. subcortical WM). In contrast to our previous observations in uncompromised preterm lambs [2], there was no apparent benefit of a protective ventilation strategy in lambs exposed to intrauterine inflammation.

### Ventilation, Oxygenation and Haemodynamics

Intra-amniotic guided LPS injection is a well-established method of causing consistent and repeatable fetal inflammatory response syndrome evidenced by pulmonary and systemic inflammation [27], [29]. We previously demonstrated that preterm lambs exposed to LPS 2 or 4 days after IA LPS and exposed to a PEEP induced haemodynamic challenge had adverse cerebral haemodynamics and increased incidence and severity of brain injury compared to control ventilated lambs [39]. The peak inflammatory response of the brain to ventilation induced brain injury occurred 2 days after IA LPS, which corresponds to the peak pulmonary and systemic cytokine response after IA LPS occuring between 24 and 72 hours after administration [29]. We previously demonstrated that a protective ventilation strategy improved respiratory outcomes, ventilation requirements and haemodynamic stability compared to an injurious high V_T_ strategy in control lambs [2]. However, in this study the same protective ventilation strategy improved only some indices of ventilation in LPS exposed preterm lambs, and did not preserve cardiopulmonary or cerebral haemodynamic stability during the transition at birth. The similarity in ventilation requirements is likely attributed to increased endogenous surfactant production in LPS exposed lambs [40], [41], thus preventing poor respiratory outcomes observed previously in control lambs that received high V_T_ ventilation [2].

The initiation of ventilation has previously been shown to increase haemodynamic instability resulting in increased vascular leakage [2], [38]. Protective ventilation after IA LPS did not prevent haemodynamic instability. While CBF and cerebral oxygen delivery were not different between groups, all ventilated lambs exhibited highly variable CBF and cerebral oxygen delivery suggesting poor cerebrovascular regulation. Variable and unstable CBF can be detrimental to a preterm infant because it increases the risk of hypoxic/ischaemic insult and possible haemorrhage, particularly in the absence of autoregulation [14], [42]. Most preterm infants <30 weeks have episodes of impaired autoregulation [43], and a link between chorioamnionitis and impaired autoregulation has been suggested in infants [44], [45] and sheep [21], although this has not been extensively studied. In this study, ventilated lambs maintained constant cerebral blood volume during the ventilation period suggesting cerebral vasoparalysis; normally there is a gradual decline in cerebral blood volume after birth as oxygen delivery increases [46]. Clinically, preterm infants exposed to chorioamnionitis have reduced variability in oxygenated and deoxygenated haemoglobin in brain tissue, with infants exhibiting the lowest variation having the most severe intraventricular haemorrhage and periventricular leukomalacia [47]. Fluctuating cerebral blood flow on a background of poor autoregulation may explain, at least in part, the increased risk of intraventricular haemorrhage in infants exposed to chorioamnionitis [48]. However, in our study, the fluctuations in CBF noted in both ventilation groups did not result in increased vascular leakage. These findings suggest that ventilation after acute intrauterine inflammation does not exacerbate vascular leakage and is not the cause of increased intraventricular haemorrhage in preterm infants exposed to choriomanionitis; more studies are required to confirm this.

The (non-statistically significant) ∼5–10% reduction in cerebral tissue oxygenation in the LPS_INJ_ lambs compared to the LPS_PROT_ lambs likely has physiological implications [49]. Animal studies show that a prolonged period of TOI less than 55% is a strong predictor of neurologic injury [50]; thus, the lower TOI noted in the LPS_INJ_ animals is indicative of an increased risk of brain injury. The time course of our study, which has focused on effects occurring immediately after birth, is insufficient for manifestation of neurologic injury. The lower TOI in LPS_INJ_ lambs is likely explained by increased cerebral oxygen extraction compared to LPS_PROT_ lambs, indicative of increased metabolic activity.

### Lung and brain inflammation and injury

Ventilation for 15 min initiates a pulmonary, systemic and cerebral inflammatory response in preterm lambs, with the intensity of the response dependent upon the initial ventilation strategy used [2], [35], [51]. In this study, ventilation, irrespective of strategy, increased lung pro-inflammatory cytokine mRNA levels in lambs born after intrauterine inflammation but there was no difference between lambs ventilated with injurious or protective strategies. This lack of effect of ventilation strategy is consistent with pulmonary inflammation responses in preterm lambs exposed to ureaplasma colonisation [52]. These studies together suggest that chorioamnionitis *per se*, and not the specific pro-inflammatory stimulus (i.e. LPS or live Ureaplasmas) is responsible. This is in contrast to our previous observations in otherwise healthy preterm lambs that showed greater lung inflammation after high V_T_ ventilation [7], [53]. It is apparent, therefore, that ventilation after intrauterine inflammation, irrespective of the strategy, significantly increases pulmonary inflammation and injury, with the response unable to be reduced by less-invasive strategies. This enhanced inflammatory response and subsequent progression of ventilation induced lung injury may explain the increased risk of bronchopulmonary dysplasia after chorioamnionitis [54].

We observed a similar increase in pro-inflammatory cytokine expression in the brain after both ventilation strategies. Ventilation of preterm lambs using high V_T_ initiates a pulmonary and systemic inflammatory cascade [10]–[12], [55]: given that pro-inflammatory cytokines can cross the blood-brain barrier [56], this systemic cascade is likely activating the cerebral release of pro-inflammatory cytokines; a response that the protective ventilation strategy has not been able to mitigate. Given the similar increase in lung pro-inflammatory cytokines in response to each ventilation strategy, it is not surprising that cerebral inflammation also increased similarly in both groups.

Immunohistochemistry demonstrated no change in microglial cell density after ventilation, in contrast to our previous observations of increased microglial density in preterm lambs ventilated with high V_T_ [2]. This suggests that acute LPS exposure is instigating tolerance rather than sensitization. There is conflicting evidence of sensitization and tolerance after LPS exposure, which is largely dependent on the timing of the LPS administration before examination. When LPS was administered 72 h before a second insult (such as hypoxic/ischemic episodes), sensitization was noted with aggravated brain injury [57], [58]. Conversely, when LPS was administered 24 h before a second insult, tolerance was noted with reduced brain injury after the second insult [59]. Thus, the 48 h timing used in this study may have induced tolerance to a second insult; in this case, ventilation.

Along with microglia, astrocytes also play a role in inflammation and have the ability to instigate inflammation and produce cytokines [60]. Thus, the increase in pro-inflammatory cytokines after ventilation could be attributed to the increased density of GFAP-positive astrocytes noted. Further, cell death was also increased in the subcortical WM after ventilation. In the normal developing brain, cell death occurs naturally as a mechanism to refine cellular connections and pathways [61]. However, in our study ventilation, irrespective of strategy, increased cell death above baseline. Importantly, the TUNEL-positive cells were found sparsely throughout the WM and were not isolated to dense focal areas indicative of key sites of injury. Further, the increase in TUNEL-positive cells following ventilation, despite its significance, was not a substantial increase which is most likely due to the early time point at which apoptosis is being assessed.

A limitation of our study is that although the effect of LPS on the brain has been well characterized, the effect of ventilation after IA LPS on brain inflammation and injury may be time dependent. We chose to assess the time of the peak fetal cytokine response to IA LPS; however, resultant alterations in the brain may not be apparent until later. Indeed, variability in timing of inflammation/infection and subsequent delivery is likely the cause of controversy surrounding the variable reports of associations between chorioamnionitis and neonatal morbidities including bronchopulmonary dysplasia, periventricular leukomalacia and intraventricular haemorrhage. A further limitation is that the duration of ventilation in our study may not have been sufficient to induce profound histological injury within the preterm brain. The duration of ventilation was chosen as it corresponds to the peak inflammatory cascade after ventilation onset [62]. Indeed, increasing the duration of ventilation in preterm infants is known to increase the risk of WM injury [63]. We compared our findings to unventilated preterm lambs to examine the influence of positive pressure ventilation versus a naïve lung. It may be more appropriate to compare lung and brain inflammation and injury to spontaneously breathing lambs, but this is not possible at this gestation, as these lambs, even with antenatal corticosteroids, cannot maintain adequate respiratory support without significant intervention. Lastly, microglia were characterized as amoeboid if they had a large, densely stained soma with completely protracted processes and all other Iba-1 positive cells were classified as ramified. This does not strictly differentiate between activated and resting microglia. Further analysis using TNF-α [64], CD68 [65] and MHC I and MHC II [66] would aid in phenotype differentiation which may have altered this interpretation, but this was beyond the scope of this study, and unlikely to impact significantly on our observed findings.

In summary, ventilation after IA LPS resulted in a profound inflammatory response within the preterm ventilated lung and within the cerebral WM, with some histological indices of brain injury observed. However, a protective ventilation strategy was unable to reduce lung or brain inflammation and injury in preterm lambs after IA LPS. These studies indicate that the preterm infant exposed to chorioamnionitis likely has increased susceptibility to ventilation induced lung and brain injury.
